# Ribosome biogenesis programs define a three-gene RBscore with prognostic relevance in bladder cancer

**DOI:** 10.3389/fimmu.2026.1810132

**Published:** 2026-04-16

**Authors:** Guangyue Luo, Weibo Wang, Supeng Tai, Lei Yan, Hailang Luo, Yifan Chang, Lexing Yang, Junyi Yan, Jun Zhou, Chaozhao Liang

**Affiliations:** 1 Department of Urology, The First Affiliated Hospital of Anhui Medical University, Hefei, Anhui, China; 2 Institute of Urology, Anhui Medical University, Hefei, Anhui, China; 3 Anhui Province Key Laboratory of Genitourinary Diseases, Anhui Medical University, Hefei, Anhui, China

**Keywords:** bladder cancer, immune responses, prognostic genes, prognostic model, ribosome biogenesis

## Abstract

**Background:**

Bladder cancer (BLCA) is clinically heterogeneous, and conventional staging does not fully capture individual risk. Ribosome biogenesis (RiBi) is implicated in cancer, but its prognostic relevance in BLCA is not well defined.

**Methods:**

Survival-annotated tumor transcriptomes from TCGA-BLCA were analyzed and externally evaluated in GSE13507. Tumor-normal differential expression was intersected with a curated RiBi gene set to prioritize candidates. Cox modeling with machine-learning–based strategy selection yielded a three-gene ribosome biogenesis–related score (RBscore). RBscore was evaluated for overall survival (OS) stratification, independence from clinicopathological variables, and integration with nodal status in a nomogram. Pathway enrichment, immune features, pharmacogenomic associations supported by molecular docking, and somatic mutation and transcription factor (TF) network features were examined. Signature gene expression was assessed in bladder cancer cell lines and paired clinical tissues. PRKDC protein expression was further evaluated by immunohistochemistry (IHC).

**Results:**

PIN4, POP4, and PRKDC comprised RBscore, which stratified OS in TCGA-BLCA and remained prognostic in GSE13507. High RBscore tumors were enriched for antigen processing and presentation and ribosome-related programs, and RBscore groups differed in immune contexture. PRKDC expression correlated with immune checkpoint-related genes including CD274, TNFRSF14, and TNFRSF25. Pharmacogenomic analyses nominated candidate compounds, with docking supporting putative binding. TP53 mutations were more frequent in the high-RBscore group. RBscore genes showed tumor-associated dysregulation in cells and tissues.

**Conclusion:**

RBscore captures prognostic heterogeneity in BLCA and connects RiBi-associated transcriptional programs with pathway activity, immune contexture, and complementary pharmacogenomic and genomic features, providing a basis for integrative risk assessment and testable hypotheses for downstream validation.

## 1 Introduction

Bladder cancer (BLCA) is the second most common malignancy of the urinary tract and remains a major contributor to cancer-related morbidity and mortality worldwide (1). By depth of invasion, BLCA is classified as non-muscle invasive or muscle invasive disease, which differ markedly in recurrence risk, progression and treatment strategies (2). Clinically, patients typically present with painless gross hematuria, sometimes accompanied by irritative lower urinary tract symptoms (3). Diagnosis is established by cystoscopy with pathologic assessment and supported by imaging for staging and surveillance (4). Non-muscle-invasive bladder cancer (NMIBC) is usually treated with transurethral resection of bladder tumor (TURBT) followed by intravesical chemotherapy, whereas muscle-invasive bladder cancer (MIBC) often necessitates radical cystectomy combined with neoadjuvant chemotherapy (5–7). Immune checkpoint blockade and fibroblast growth factor receptor (FGFR)-targeted therapy have expanded options for selected patients (8, 9). However, recurrence and progression remain common, consistent with pronounced biological heterogeneity that complicates patient-level risk assessment and treatment selection (10). The molecular programs that drive this heterogeneity are incompletely resolved, and clinically actionable biomarkers that link tumor biology to individual risk remain limited (11).

Ribosome biogenesis (RiBi) produces ribosomes to sustain protein synthesis (12). This coordinated program comprises rRNA transcription and processing, ribosomal protein production, nucleolar assembly of preribosomal particles and maturation into functional ribosomes (13). Beyond housekeeping, RiBi interfaces with growth control and stress responses (14, 15). In cancer, RiBi dysregulation has been linked to altered translational output and nucleolar stress signaling, and it can intersect with replication stress and DNA damage responses, often in relation to the p53 axis (16). Mechanistically, ribosome biogenesis has been connected to tumor suppression via the 5S RNP-MDM2-p53 pathway, and perturbation of this axis has been associated with metabolic and immune contexture changes in cancer (17, 18). Despite these insights, the prognostic relevance of ribosome biogenesis–related genes (RBRGs) in BLCA, and their relationships to immune contexture and therapeutic response remain incompletely defined.

In this study, we analyzed survival-annotated tumor transcriptomes to derive a three-gene ribosome biogenesis–related score (RBscore) for risk stratification in bladder cancer. RBscore was evaluated in an independent cohort and linked to pathway activity, immune features, genomic correlates, and drug-response signals. Expression of key genes was assessed in bladder cancer cell lines and clinical tissues. Together, these analyses position ribosome biogenesis programs as an interpretable axis of BLCA heterogeneity and generate testable hypotheses for downstream mechanistic and translational studies.

## 2 Materials and methods

### 2.1 Data acquisition

Transcriptomic profiles and corresponding clinical information for BLCA were obtained from The Cancer Genome Atlas (TCGA) database via UCSC Xena browser (https://xenabrowser.net/). The TCGA cohort comprised 426 samples, including 407 tumor tissues and 19 normal tissues, and served as the training cohort with complete survival outcomes. The GSE13507 dataset was downloaded from the Gene Expression Omnibus (GEO) database (https://www.ncbi.nlm.nih.gov/geo/) and used as an external validation cohort. This dataset included 165 primary BLCA samples, 23 recurrent BLCA samples, 58 adjacent mucosal tissues and 10 normal bladder tissues. A curated list of 331 RBRGs was obtained from a published study and used for downstream analyses (19).

### 2.2 Candidate gene identification and functional annotation

Differential expression analysis between BLCA and normal tissues in the training cohort was performed using the DESeq2 R package. Genes with an absolute log2 fold change (|log2FC|) > 0.5 and false discovery rate (FDR) < 0.05 were defined as differentially expressed genes (DGEs). Functional enrichment analysis was performed using the clusterProfiler R package, including Kyoto Encyclopedia of Genes and Genomes (KEGG) pathway enrichment and Gene Ontology (GO) analyses. A protein–protein interaction network was constructed using the STRING database (https://string-db.org) with a minimum interaction score of 0.90.

### 2.3 Prognostic gene selection and RBscore construction

Candidate gene expression profiles were integrated with overall survival (OS) time and status data to generate survival-annotated datasets for the training and validation cohorts. In the training cohort, candidate genes associated with OS were first screened using univariate Cox proportional hazards regression implemented in the survival R package. The proportional hazards (PH) assumption was then assessed, and genes without evidence of violation were retained for downstream modeling (PH test, P > 0.05). Hazard ratios (HRs) and 95% confidence intervals (CIs) from the univariate analyses were visualized using forest plots generated with the forestplot R package.

The training and validation cohorts were generated from different expression platforms, namely RNA-seq in TCGA-BLCA and microarray in GSE13507. To improve cross-platform comparability, candidate gene expression values were standardized as Z-scores before machine-learning analysis. We then combined 10 machine-learning algorithms into 95 modeling strategies and used the concordance index (C-index) as the primary performance metric. Model selection was based on performance in the validation cohort, while also considering discrimination in the training cohort and the mean C-index across both cohorts. On this basis, StepCox[both] + RSF was selected as the optimal strategy.

This strategy involved sequential feature selection followed by survival modeling. Bidirectional stepwise Cox regression based on the Akaike information criterion (AIC) was first used to identify the subset of genes that best balanced model fit and parsimony. Backward elimination was then applied to remove variables without statistical support. Through this procedure, PIN4, POP4, and PRKDC were retained as the final prognostic genes. These three genes were subsequently entered into a random survival forest (RSF) model with ntree = 1000, nodesize = 5, mtry = 5, and splitrule = “logrank”. After model training, variable importance (VIMP) was calculated for each gene, and all three genes showed positive VIMP values, supporting their contribution to model prediction. Based on this final StepCox[both] + RSF framework, we constructed a RiBi-based prognostic signature termed RBscore. For each patient, RBscore was defined as the individual risk value predicted by the trained RSF model using the standardized expression values of PIN4, POP4, and PRKDC as input features.

### 2.4 Prognostic performance of RBscore

Model discrimination was assessed in the training and validation cohorts using time-dependent receiver operating characteristic (ROC) curves for 1-, 3-, and 5-year OS. Patients were stratified into high- and low-RBscore groups using cohort specific cutoffs determined by maximally selected log-rank statistics, and survival differences between risk strata were evaluated using Kaplan-Meier curves. RBscore distribution plots were used to summarize RBscore values alongside event status.

### 2.5 Prognostic nomogram construction

In the training cohort, univariate Cox regression was used to evaluate associations of clinical variables and RBscore with OS. Variables with *P* < 0.05 in univariate analysis and no evidence of proportional hazards violation were entered into multivariable Cox regression to identify independent prognostic factors. Independent factors were integrated into a nomogram constructed using the rms R package to estimate 1-, 3-, and 5-year OS. Nomogram performance was evaluated using calibration curves and time-dependent ROC curves.

### 2.6 Differential expression and subgroup survival analysis

Differential expression analysis was performed between the high- and low-RBscore groups in the training cohort, and genes were ranked by log2FC to generate an ordered list for enrichment analysis. Gene set enrichment analysis (GSEA) was then conducted using the c2.kegg.v7.4.symbols gene set from the Molecular Signatures Database (MSigDB, http://www.gsea-msigdb.org/gsea/msigdb/index.jsp). In parallel, subgroup Kaplan-Meier curves were generated within strata defined by clinical characteristics to evaluate RBscore performance, and survival differences between RBscore groups were assessed using log-rank tests.

### 2.7 Immune infiltration and checkpoint analysis

Immune cell infiltration in the training cohort was estimated using the CIBERSORT algorithm to quantify the relative fractions of 22 immune cell types. Samples with a permutation-based CIBERSORT *P* < 0.05 were retained for downstream analyses. Differences in immune cell fractions between high- and low-RBscore groups were assessed using the Wilcoxon rank-sum test. Based on these differential immune features, Spearman’s correlation was used to characterize immune cell co-infiltration patterns and to evaluate associations between prognostic gene expression and differentially infiltrating immune cells. Tumor microenvironment features were further profiled using the ESTIMATE R package to derive immune score, stromal score, ESTIMATE score and tumor purity. Immune checkpoint analyses were conducted to examine associations between prognostic genes and checkpoint expression and to compare checkpoint expression between RBscore strata.

### 2.8 Drug response prediction and molecular docking

Drug sensitivity data for the NCI-60 cancer cell lines were obtained from the CellMiner database (https://discover.nci.nih.gov/cellminer/home.do). Prognostic gene expression was integrated with drug activity profiles, and Pearson correlation was used to assess gene-drug associations. To estimate predicted patient level drug response, a panel of 138 chemotherapeutic and targeted agents was obtained from the Cancer Therapeutics Response Portal (https://www.cancerrxgene.org/), and IC50 values were inferred in the training cohort using the pRRophetic R package. Predicted IC50 values were compared between RBscore strata using the Wilcoxon rank-sum test. For selected compound-target pairs, molecular docking was performed to evaluate binding potential. Protein structures were retrieved from the Protein Data Bank (https://www.rcsb.org/), and compound structures were obtained from the PubChem (https://pubchem.ncbi.nlm.nih.gov) and prepared using OpenBabel software. Receptor proteins were prepared in PyMOL software by removing water molecules and co-crystallized ligands and by adding hydrogen atoms. Docking inputs were generated using AutoDock Tools software.

### 2.9 Somatic mutation analysis

Somatic mutation data for BLCA were obtained from the training cohort. Mutation profiles were summarized using the maftools R package. Tumor mutational burden was calculated as mutations per megabase and compared between high- and low-RBscore groups using the Wilcoxon rank-sum test.

### 2.10 Regulatory networks construction

To characterize upstream regulatory features of the prognostic genes, putative transcription factor (TF) regulators were inferred using ENCODE-derived TF-target interaction data as implemented in NetworkAnalyst (https://www.networkanalyst.ca/). Predicted TF–gene interactions were integrated to construct a regulatory network and visualized in Cytoscape software.

### 2.11 Cell culture

Human bladder urothelial SV-HUC-1 cells (Procell, China) and bladder cancer T24 cells (Procell, China) were cultured in RPMI-1640 medium (Gibco, USA) supplemented with 10% fetal bovine serum (FBS; Gibco, USA) and 1% penicillin–streptomycin at 37 °C in a humidified incubator with 5% CO_2_. Cells were passaged at 70% to 80% confluence and harvested during logarithmic growth for RNA and protein extraction.

### 2.12 RT–qPCR

Total RNA was extracted using TRIzol reagent (Invitrogen, USA). The extracted RNA was treated with dsDNase before reverse transcription. cDNA was synthesized using the HiScript II First Strand cDNA Synthesis Kit (Vazyme, China). qPCR was performed using AceQ U+ Probe Master Mix (Vazyme, China) on a real-time PCR system. Relative mRNA expression of PIN4, POP4, and PRKDC was calculated using the 2^-ΔΔCt^ method with β-actin (ACTB) as the internal reference. Primer and probe sequences are provided in **Supplementary Table 1**. All reactions were run in triplicate.

### 2.13 Western blot analysis

Total protein was extracted using ice-cold RIPA lysis buffer supplemented with protease inhibitors. Protein concentration was determined using a BCA assay. Equal amounts of protein were separated by SDS-PAGE and transferred to PVDF membranes (Millipore, USA). Membranes were blocked with 5% nonfat milk and incubated overnight at 4 °C with primary antibodies against POP4 (Proteintech, China), PIN4 (Affinity, China), PRKDC (Affinity, China), and GAPDH (Affinity, China). After incubation with HRP-conjugated secondary antibodies (Zsbio, China), signals were detected using an enhanced chemiluminescence substrate and quantified by densitometry. Protein abundance was normalized to GAPDH.

### 2.14 Clinical specimens

We conducted a cross-sectional analysis to examine the association between PRKDC expression and clinicopathological features in BLCA. The analysis included 76 patients with MIBC who underwent radical cystectomy between January 2024 and September 2025 at the High-tech Campus of the First Affiliated Hospital of Anhui Medical University. PRKDC expression was assessed by immunohistochemistry (IHC) in tumor and adjacent normal tissues from all 76 patients. In addition, fresh specimens from the most recently collected five cases were analyzed by RT–qPCR to evaluate mRNA expression. Written informed consent was obtained from all participants, and the study protocol was approved by the Ethics Committee of the First Affiliated Hospital of Anhui Medical University.

### 2.15 IHC

Tissue specimens were fixed in 4% paraformaldehyde, paraffin-embedded, and sectioned. After deparaffinization and antigen retrieval, sections were blocked and incubated with antibodies against PRKDC (Affinity, China), followed by HRP-DAB development and hematoxylin counterstaining. Slides were independently evaluated by two pathologists blinded to clinical information, with discrepancies resolved by consensus. Staining was semi-quantified, and IHC scoring was performed using the Remmele immunoreactive score (IRS), defined as the product of the proportion (0-4) and intensity (0-3) scores (range, 0-12).

### 2.16 Statistical analysis

All statistical analyses were performed in R (version 4.2.2). Unless otherwise specified, two-group comparisons were conducted using a two-sided Wilcoxon rank-sum test. Correlations between variables were assessed using Pearson’s or Spearman’s correlation, as appropriate. Categorical variables were compared using Fisher’s exact test. Survival differences were evaluated using Kaplan-Meier curves with the log-rank test. A *P* value < 0.05 was considered statistically significant.

## 3 Results

### 3.1 Differential expression and functional enrichment of RBRGs in BLCA

**Figure 1:**
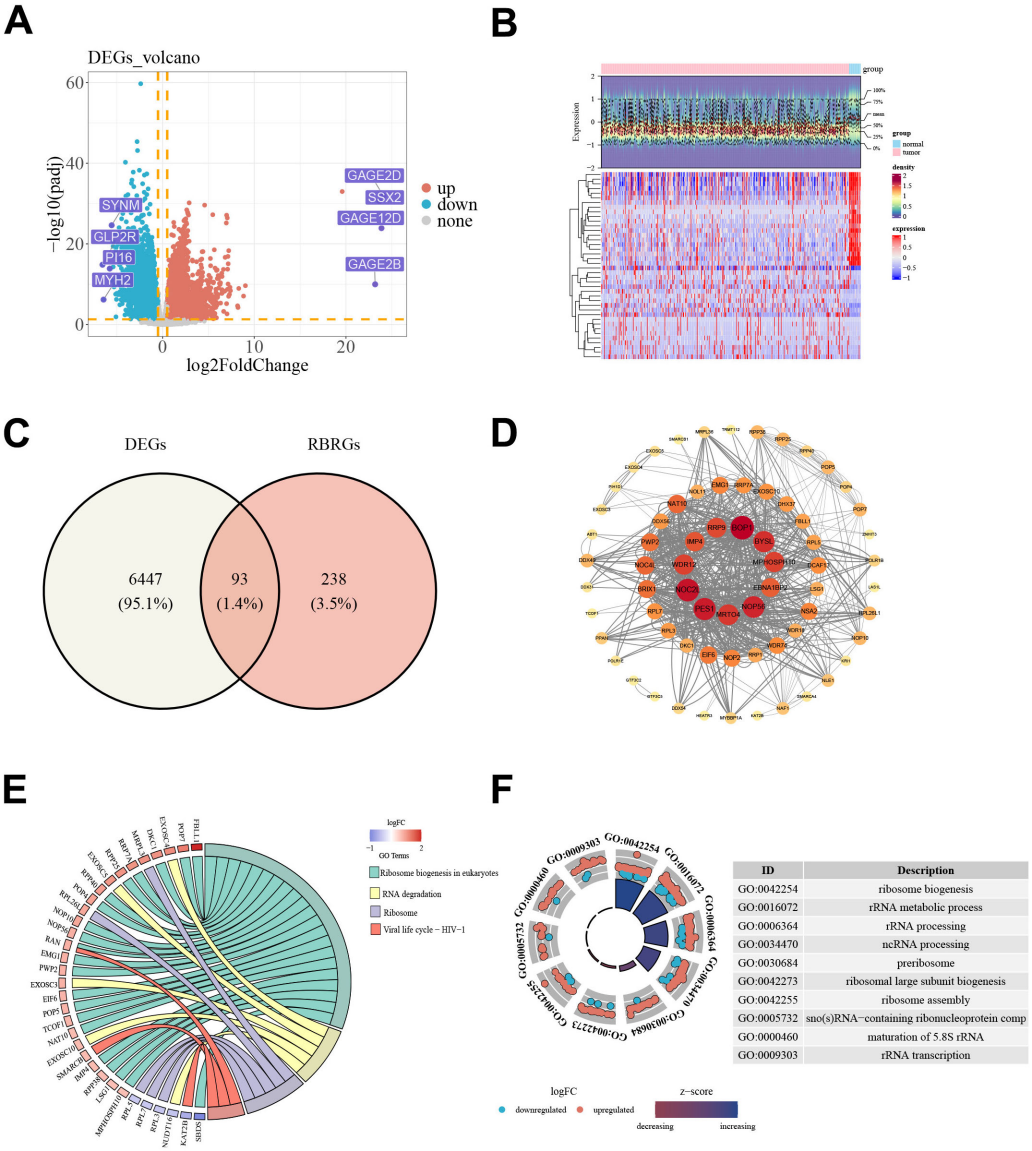
Identification and functional characterization of RBRGs in BLCA. **(A)** Volcano plot of DEGs between BLCA tumor and normal bladder tissues in TCGA-BLCA. **(B)** Expression patterns of DEGs across BLCA tumor and normal bladder samples. **(C)** Venn diagram showing the overlap between DEGs and the curated RBRG set. **(D)** PPI network of candidate genes. **(E)** KEGG enrichment analysis of candidate genes. **(F)** GO enrichment analysis of candidate genes. BLCA, bladder cancer; DEGs, differentially expressed genes; RBRGs, ribosome biogenesis-related genes; PPI, protein-protein interaction; KEGG, Kyoto Encyclopedia of Genes and Genomes; GO, Gene Ontology.

At first, differential expression analysis was performed between BLCA tumor tissues and normal bladder tissues in the TCGA training cohort, identifying 6,540 differentially expressed genes, including 3,748 upregulated and 2,792 downregulated genes (**Figures 1A, B**). Intersecting these DEGs with the reported RBRG set yielded 93 candidate genes for subsequent analyses (**Figure 1C**). The protein-protein interaction (PPI) network of these candidates contained 68 nodes and 736 edges (**Figure 1D**). KEGG enrichment highlighted four pathways, including ribosome biogenesis in eukaryotes, RNA degradation, ribosome, and Viral life cycle-HIV-1 (**Figure 1E**). Gene Ontology enrichment yielded 228 significant terms, comprising 137 biological processes, 36 cellular components, and 55 molecular functions, and the top terms mapped to RiBi-related processes, including ribosome biogenesis, rRNA metabolic and processing terms, and ribosome assembly (**Figure 1F**).

### 3.2 Establishment and validation of an RiBi-based signature

**Figure 2:**
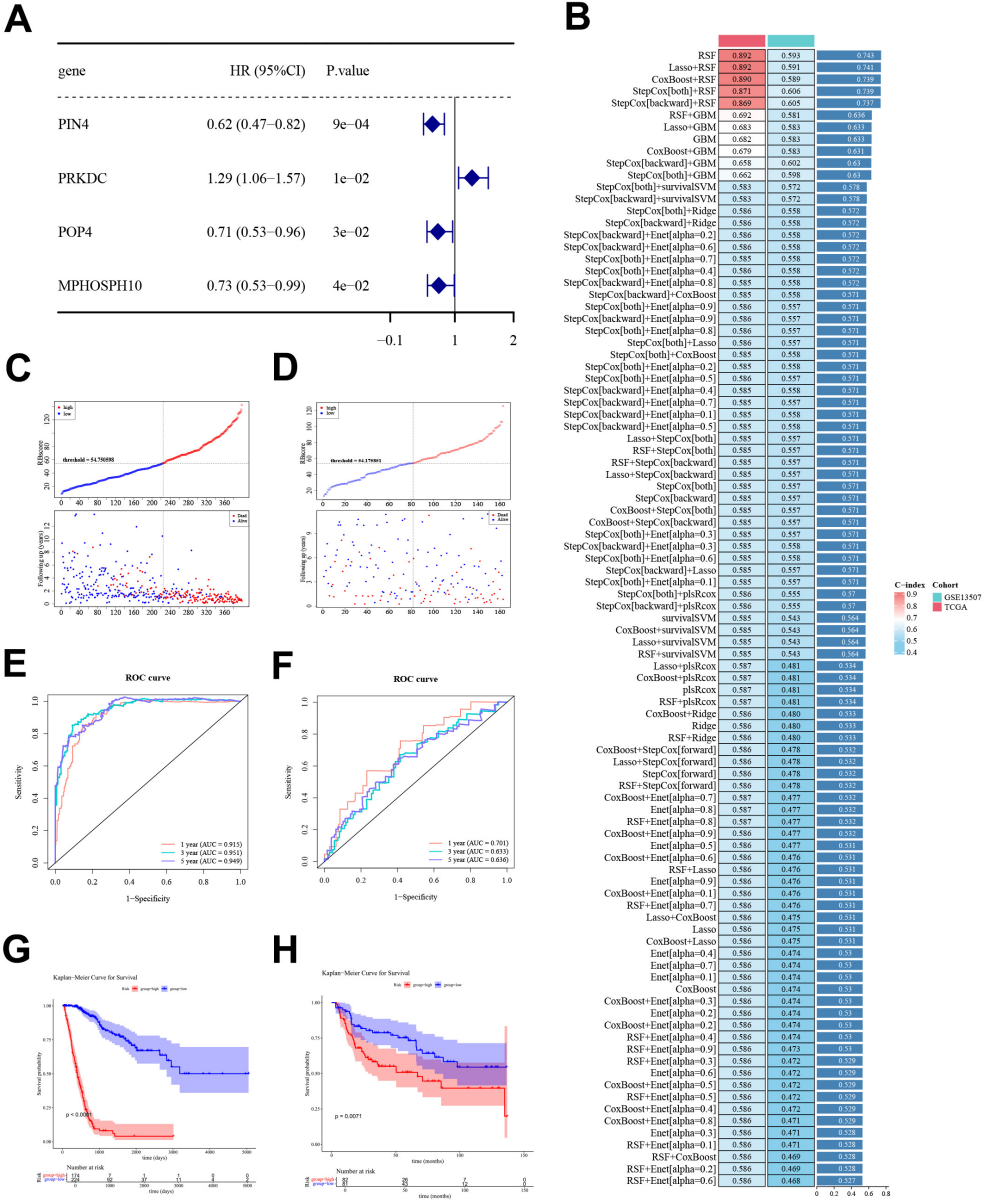
Prognostic gene selection and RBscore construction in BLCA. **(A)** Forest plot of univariate Cox regression results for PIN4, POP4, PRKDC, and MPHOSPH10. **(B)** Performance of modeling strategies in the training and validation cohorts, summarized by C-index. **(C, D)** RBscore distribution and event status in the training and validation cohorts. **(E, F)** Time-dependent ROC curves for OS prediction in the training and validation cohorts. **(G, H)** Kaplan-Meier curves for OS in high- and low-RBscore groups in the training and validation cohorts. BLCA, bladder cancer; RBscore, ribosome biogenesis–related score; OS, overall survival; ROC, receiver operating characteristic; PH, proportional hazards; HR, hazard ratio; CI, confidence interval; C-index, concordance index.

Candidate gene expression profiles were linked to OS data, yielding 398 cases in the training cohort and 163 cases in the external validation cohort with complete matched information. Univariate Cox regression identified seven genes associated with OS in the training cohort (*P* < 0.05; **Figure 2A**). The PH assumption was further evaluated, and four genes were retained for downstream modeling, including PIN4, PRKDC, POP4 and MPHOSPH10 (PH test *P* > 0.05; **Supplementary Figure 1**). PRKDC was associated with increased mortality risk, whereas PIN4, POP4 and MPHOSPH10 were associated with reduced risk (**Figure 2A**). Among 95 modeling strategies, StepCox[both]+RSF achieved the best overall performance, with a C-index of 0.871 in the training cohort and 0.606 in the validation cohort (**Figure 2B**). PIN4, POP4, and PRKDC were retained as the final prognostic genes and used to derive the prognostic signature, RBscore, for subsequent analyses. RBscore stratified patients into high and low groups in both cohorts, using cutoffs of 54.750598 in the training cohort and 54.175851 in the validation cohort (**Figures 2C,D**). Time-dependent ROC curves for OS indicated strong discrimination in the training cohort, with areas under the curve (AUCs) of 0.915, 0.951, and 0.949 at 1-, 3-, and 5-year time points, respectively. Discrimination was more modest in the validation cohort, with corresponding AUCs of 0.701, 0.633, and 0.636 (**Figures 2E, F**). RBscore stratification separated OS on Kaplan-Meier curves in both cohorts, with poorer OS in the high-RBscore group in the training cohort (*P* < 0.0001) and the validation cohort (*P* = 0.0071; **Figures 2G, H**).

### 3.3 RBscore is an independent prognostic factor and supports nomogram construction in BLCA

**Figure 3:**
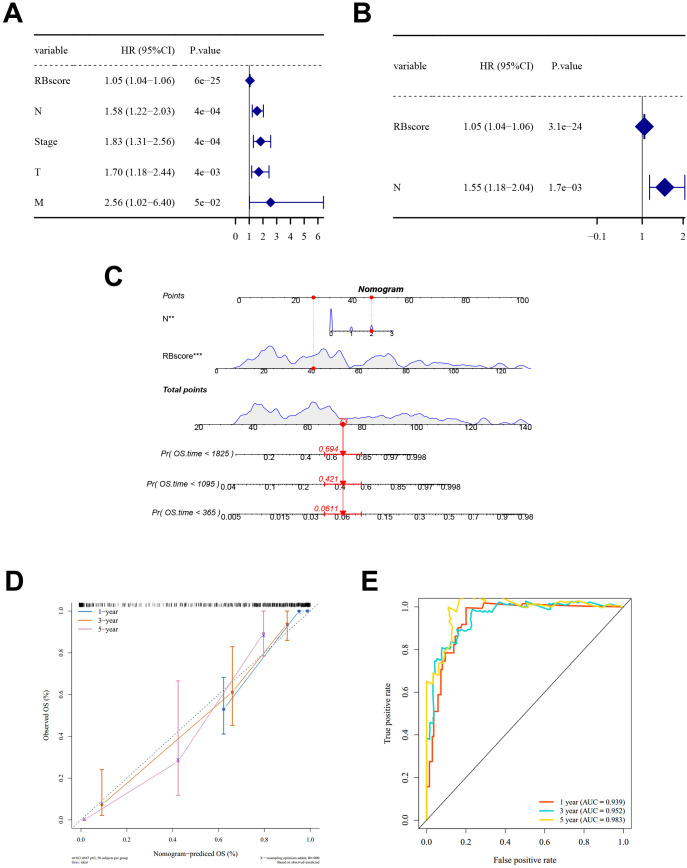
Independent prognostic value and nomogram construction of the risk model in BLCA. **(A)** Forest plot of univariate Cox regression for RBscore and clinicopathological variables. **(B)** Forest plot of multivariable Cox regression identifying independent prognostic factors. **(C)** Nomogram integrating RBscore and N stage to estimate 1-, 3-, and 5-year OS. **(D)** Calibration curves for 1-, 3-, and 5-year OS. **(E)** Time-dependent ROC curves for nomogram-predicted OS. BLCA, bladder cancer; RBscore, ribosome biogenesis-related score; OS, overall survival; ROC, receiver operating characteristic; HR, hazard ratio; CI, confidence interval.

Univariate Cox regression showed that RBscore and clinicopathological variables, including T, N, M, and overall stage, were associated with OS (*P* < 0.05, **Figure 3A**; **Supplementary Table 2**). In multivariable analysis, RBscore remained independently associated with OS, and N stage was retained as an additional independent factor (**Figure 3B**). A nomogram incorporating RBscore and N stage was constructed to estimate 1-, 3- and 5-year OS probabilities (**Figure 3C**). Calibration curves showed good agreement between predicted and observed OS (**Figure 3D**). Time-dependent ROC curves for nomogram-predicted OS showed AUCs of 0.939, 0.952, and 0.983 at 1-, 3-, and 5-year time points, respectively (**Figure 3E**).

### 3.4 Distinct pathway enrichment and consistent subgroup survival separation across RBscore strata

**Figure 4:**
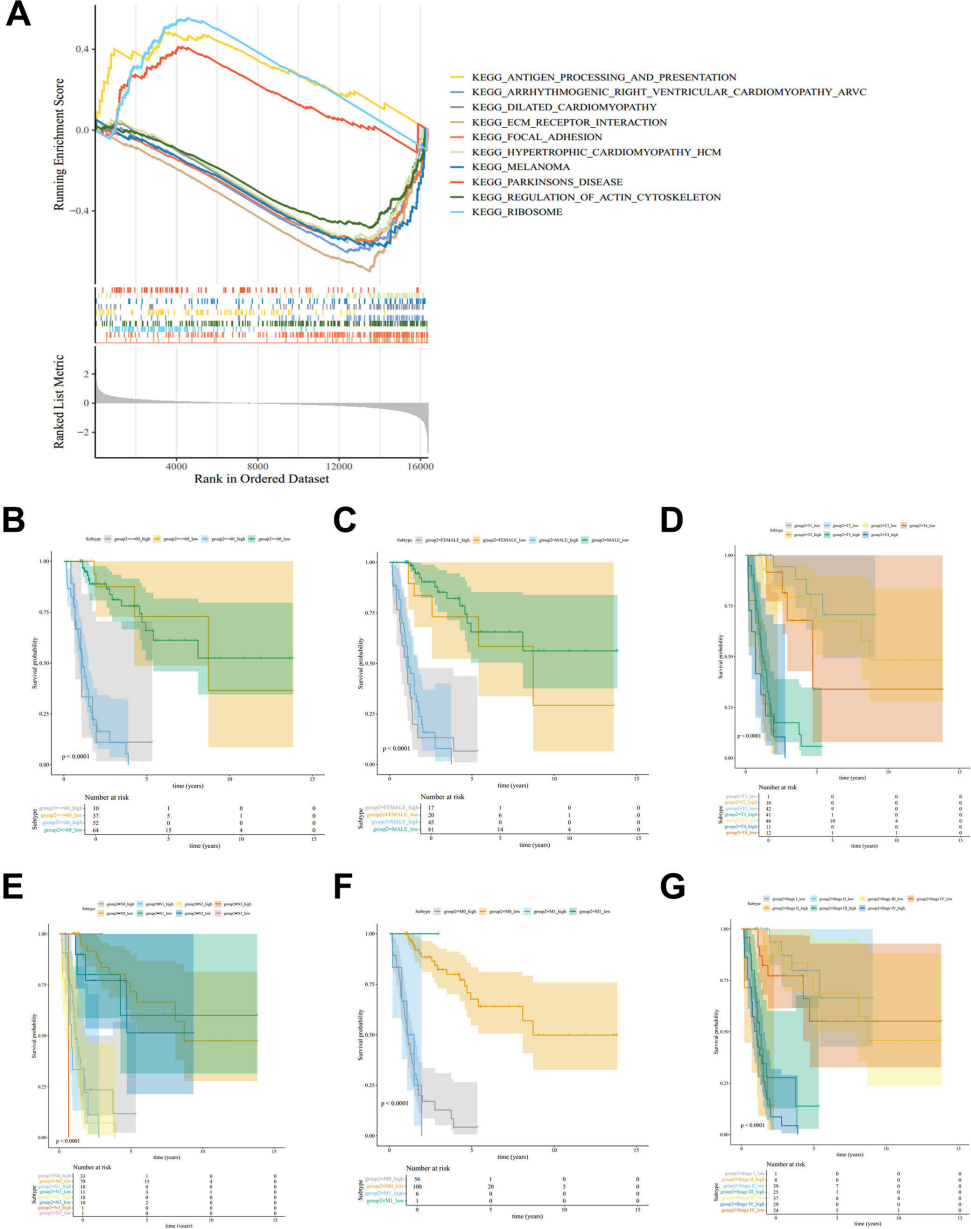
Pathway enrichment and subgroup survival patterns across RBscore strata in BLCA. **(A)** GSEA indicated differential enrichment of KEGG pathways between high- and low-RBscore groups. **(B–G)** Kaplan-Meier curves for OS in high- and low-RBscore groups within strata defined by age **(B)**, sex **(C)**, T stage **(D)**, N stage **(E)**, M stage **(F)**, and overall stage **(G)**. BLCA, bladder cancer; RBscore, ribosome biogenesis-related score; OS, overall survival; GSEA, gene set enrichment analysis; KEGG, Kyoto Encyclopedia of Genes and Genomes.

We next examined pathway-level differences and subgroup patterns across RBscore strata. GSEA indicated enrichment of KEGG antigen processing and presentation and KEGG ribosome in the high-RBscore group (**Figure 4A**). To assess whether the RBscore stratification remained informative across clinicopathological subgroups, we examined OS differences between high- and low-RBscore groups within strata defined by age, sex, overall stage, and TNM stage categories. Across these subgroups, the high-RBscore group consistently showed poorer OS than the low group within the training cohort (*P* < 0.001; **Figures 4B–G**).

### 3.5 Immune landscape variation across RBscore strata

**Figure 5:**
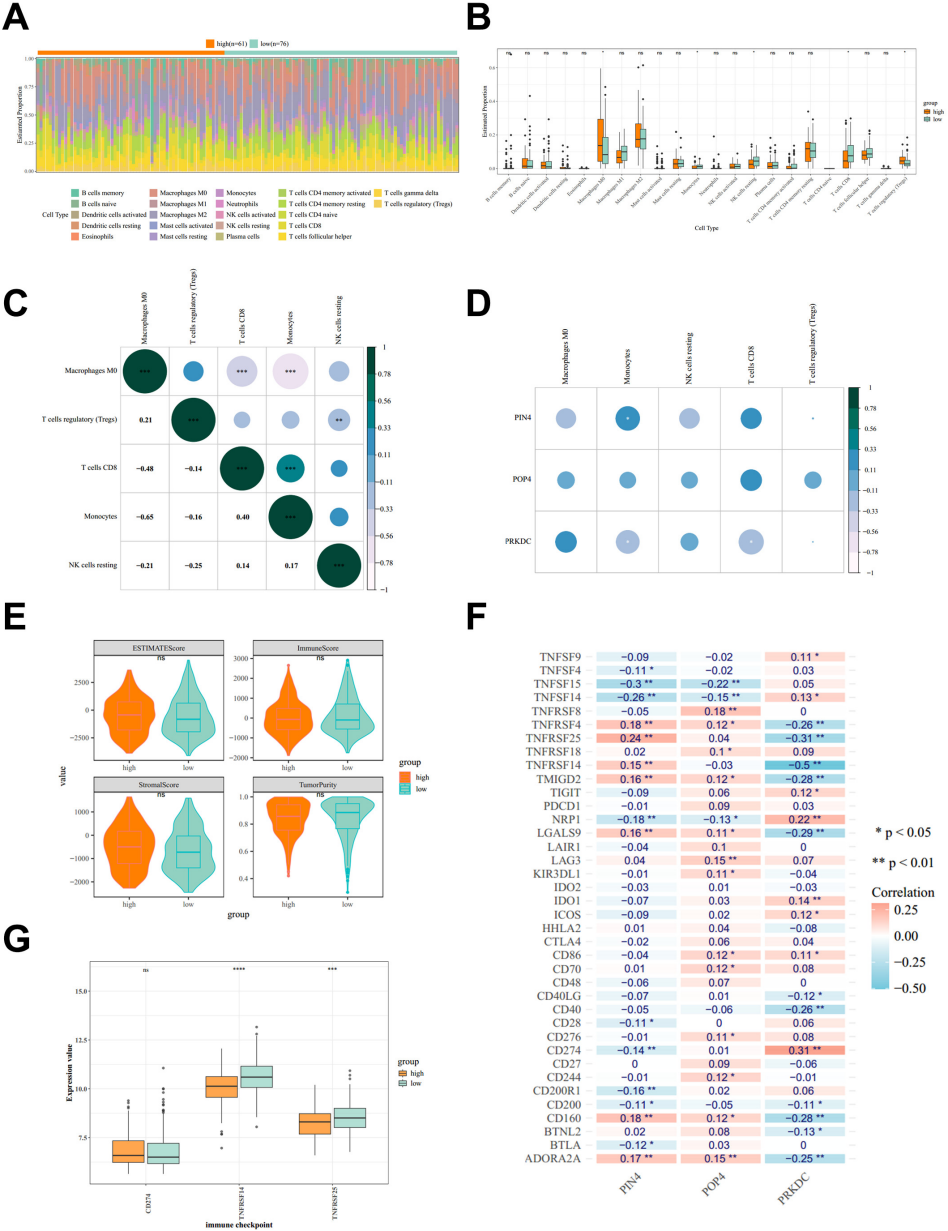
Immune landscape and immune checkpoint features across RBscore strata in BLCA. **(A)** CIBERSORT-inferred fractions of 22 immune cell types in high- and low-RBscore groups. **(B)** Comparison of immune cell fractions between high- and low-RBscore groups. **(C)** Correlation matrix of differentially represented immune cell subsets. **(D)** Correlations between RBscore signature genes and differentially represented immune cell subsets. **(E)** ESTIMATE-derived tumor purity, immune score, stromal score and ESTIMATE score across RBscore strata. **(F)** Heatmap of correlations between RBscore signature genes and immune checkpoint genes. **(G)** Expression of selected immune checkpoint genes across RBscore strata. *P < 0.05, **P < 0.01, ***P < 0.001. BLCA, bladder cancer; RBscore, ribosome biogenesis-related score.

We compared immune cell infiltration between the high- and low-RBscore groups to characterize immune features associated with RBscore stratification. The CIBERSORT algorithm produced reliable deconvolution outputs for 137 samples (permutation-based *P* < 0.05), which were retained for downstream comparisons (**Figure 5A**). Five immune cell populations differed between RBscore groups, including M0 macrophages, monocytes, resting NK cells, CD8(+) T cells and regulatory T cells (*P* < 0.05; **Figure 5B**). Among these cells, monocytes were inversely correlated with M0 macrophages (r = -0.65, *P* < 0.001) and positively correlated with CD8(+) T cells (r = 0.40, *P* < 0.001; **Figure 5C**). We next examined associations between prognostic genes and these differential immune components. PIN4 expression was negatively correlated with monocytes (r = -0.32, *P* < 0.05), whereas PRKDC expression was positively correlated with monocytes and CD8(+) T cells (r = 0.31, *P* < 0.05; **Figure 5D**). In contrast, ESTIMATE-derived tumor purity, immune score, stromal score and ESTIMATE score did not differ between RBscore groups (**Figure 5E**). Immune checkpoint associations were evaluated for prognostic genes, and three checkpoint genes were prioritized for PRKDC, including CD274, TNFRSF25 and TNFRSF14. PRKDC expression was positively correlated with CD274 (r = 0.31, *P* < 0.01) and negatively correlated with TNFRSF25 (r = -0.31, *P* < 0.01) and TNFRSF14 (r = -0.50, *P* < 0.01; **Figure 5F**). TNFRSF25 and TNFRSF14 were expressed at higher levels in the low-RBscore group (*P* < 0.001; **Figure 5G**).

### 3.6 RBscore signature gene-drug associations and predicted drug response

**Figure 6:**
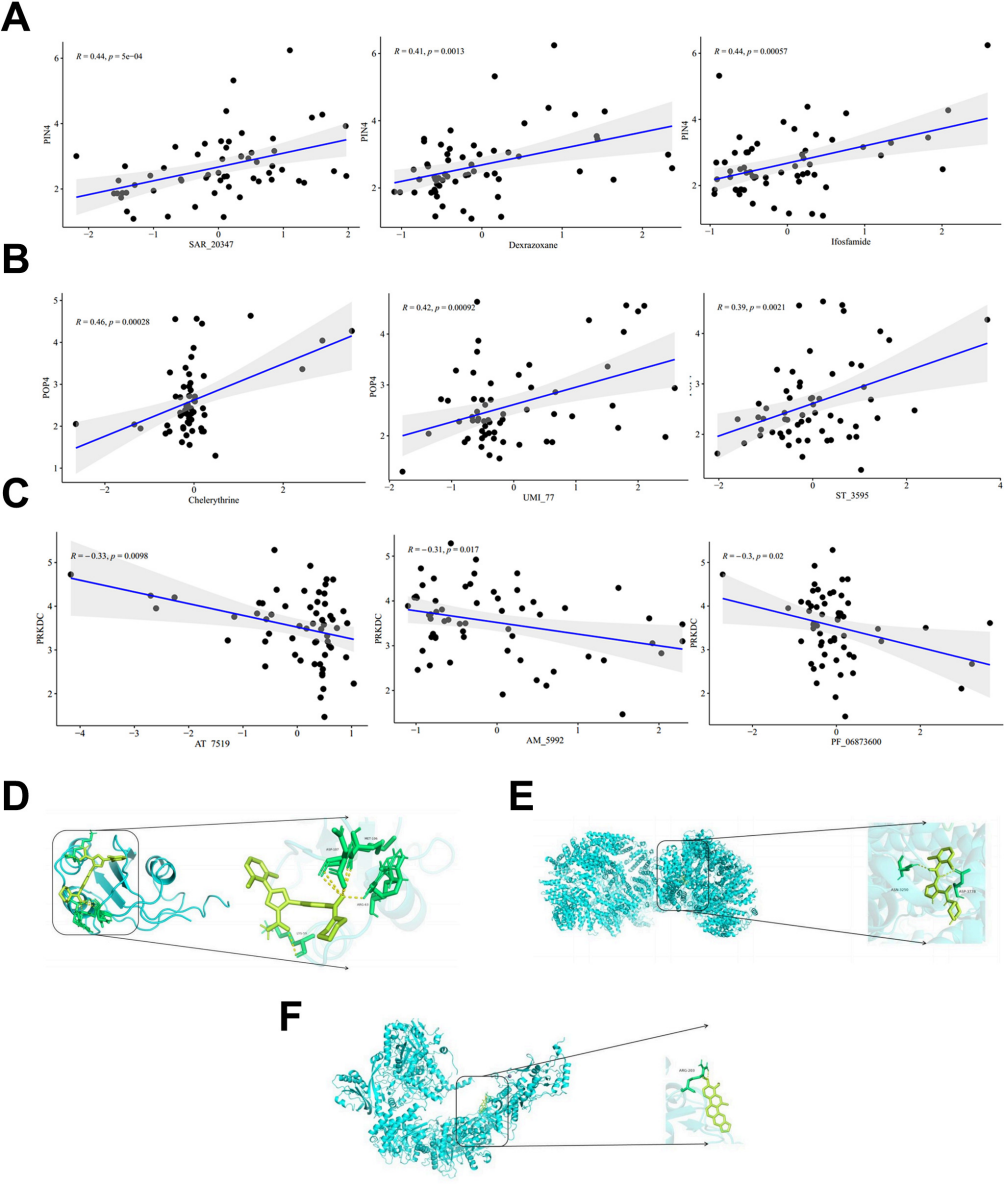
Drug sensitivity correlations and molecular docking validation of prognostic genes in BLCA. **(A–C)** Associations between PIN4, POP4, or PRKDC expression and drug activity in the NCI-60 panel. **(D–F)** Representative docking poses for SAR-20347 with PIN4 **(D)**, chelerythrine with POP4 **(E)**, and AT-7519 with PRKDC **(F)**. BLCA, bladder cancer; RBscore, ribosome biogenesis-related score.

Drug sensitivity associations were evaluated by integrating prognostic gene expression with activity profiles in the NCI-60 panel. With thresholds ofr| > 0.30 and *P* < 0.05, PIN4, POP4, and PRKDC were associated with 15, 24, and 4 compounds, respectively. Representative compounds included dexrazoxane, ifosfamide and SAR-20347 for PIN4, ST-3595, UMI-77 and chelerythrine for POP4, and AT-7519, AM-5992, and PF-06873600 for PRKDC. The strongest correlations were observed for PIN4 with SAR-20347 (r = 0.44, *P* < 0.0001), POP4 with chelerythrine (r = 0.46, *P* = 0.0003), and PRKDC with AT-7519 (r = -0.33, *P* = 0.0098; **Figures 6A–C**), and these three top-ranked gene-compound pairs were subsequently taken forward for molecular docking. All three docked pairs showed binding energies below -5 kcal/mol, supporting favorable binding in silico (**Figures 6D, F**; **Supplementary Table 3**). We next evaluated predicted patient-level drug response in the training cohort. In total, 38 compounds showed different inferred IC50 values between the high- and low-RBscore groups (*P* < 0.05, **Supplementary Figure 2**).

### 3.7 Somatic mutation patterns across RBscore strata and inferred TFs

**Figure 7:**
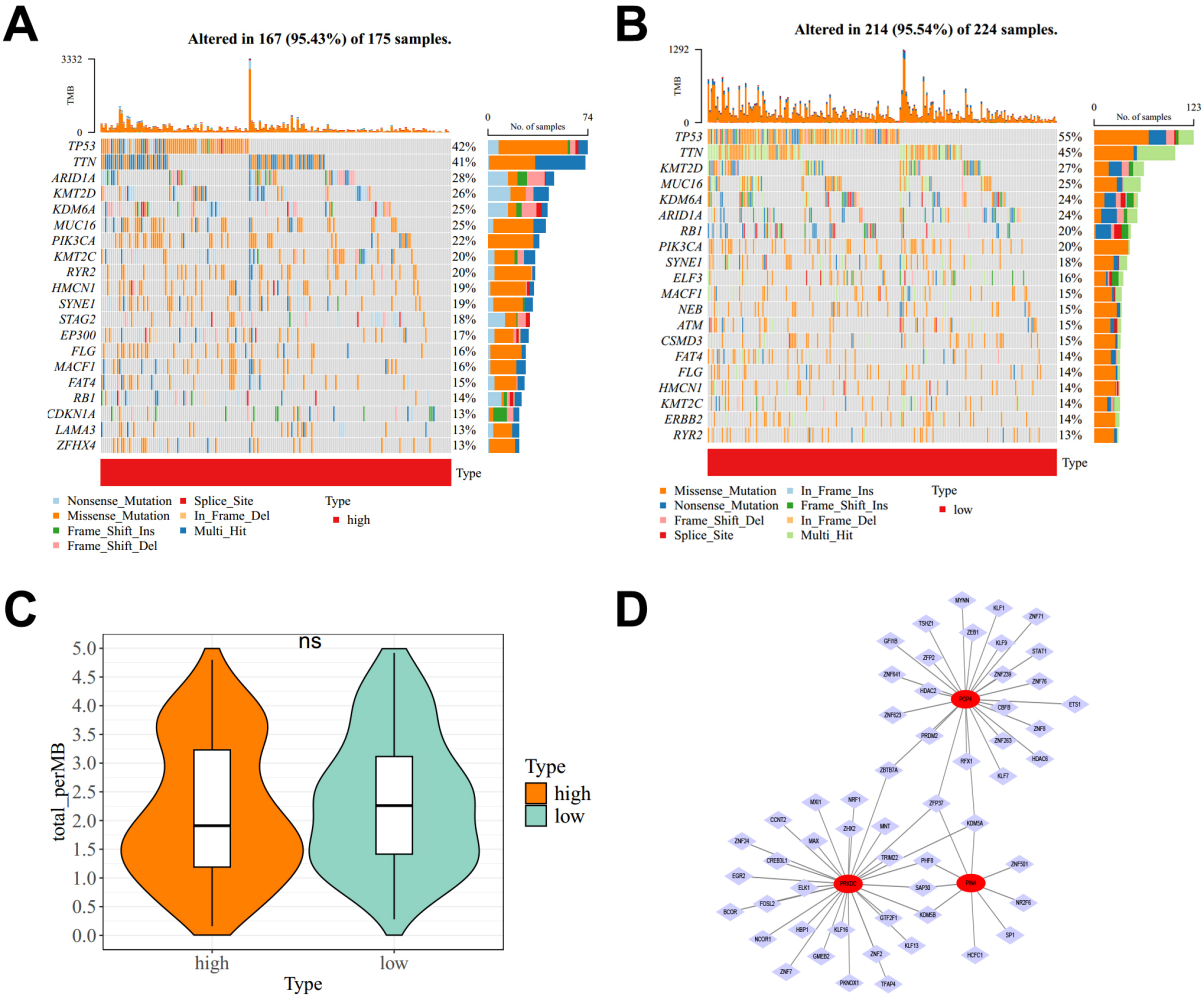
Somatic mutation patterns and inferred TF-gene network in BLCA. **(A, B)** Mutation landscapes of the low-RBscore **(A)** and high-RBscore **(B)** groups. **(C)** Tumor mutational burden in high- and low-RBscore groups. **(D)** TF-gene regulatory network connecting predicted TF regulators to RBscore signature genes. TF, transcription factor; BLCA, bladder cancer; RBscore, ribosome biogenesis-related score.

We profiled somatic mutation patterns in the high- and low-RBscore groups. Among the 20 most frequently altered genes, TP53 was the most commonly mutated, with mutation frequencies of 55% in the high-RBscore group and 42% in the low-RBscore group (**Figures 7A, B**). Tumor mutational burden did not differ between RBscore groups (**Figure 7C**). To explore upstream regulatory features of the RBscore genes, ENCODE-based TF inference suggested 9 TFs for PIN4, 25 TFs for POP4, and 29 TFs for PRKDC. Two TFs, KDM5A and ZFP37, were shared across all three genes. These interactions were integrated into a TF-gene regulatory network comprising 58 nodes and 63 edges (**Figure 7D**).

### 3.8 RBscore signature genes are dysregulated in cell lines and clinical specimens

**Figure 8:**
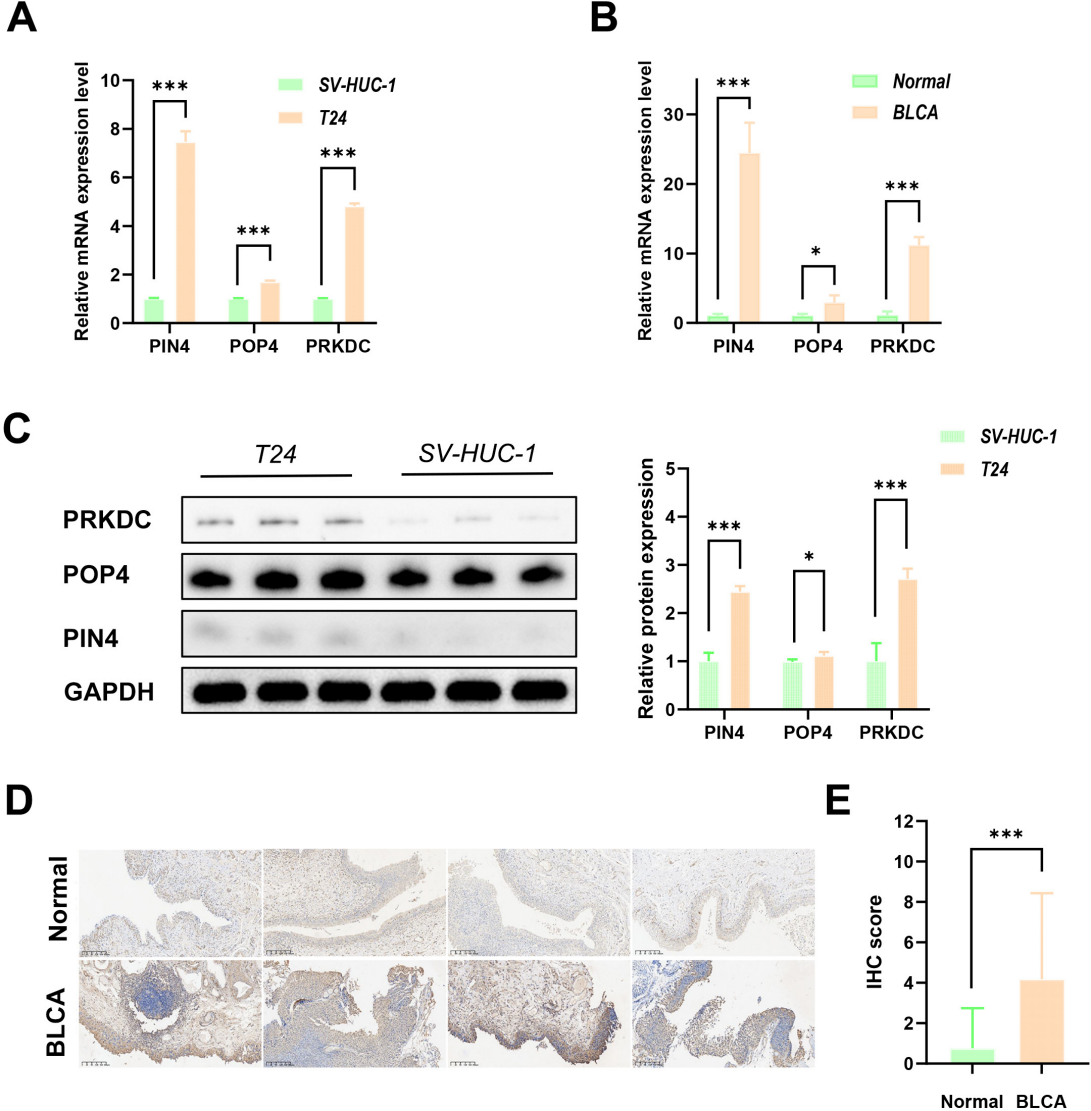
Experimental validation of RBscore signature gene expression. **(A)** RT-qPCR of PIN4, POP4, and PRKDC mRNA in SV-HUC-1 and T24 cells. **(B)** RT-qPCR of PIN4, POP4, and PRKDC mRNA in paired BLCA and adjacent normal tissues. **(C)** Western blot analysis and quantification of PIN4, POP4, and PRKDC protein in SV-HUC-1 and T24 cells. **(D)** Representative IHC images of PRKDC in BLCA and adjacent normal tissues. **(E)** Quantification of PRKDC IHC scores in BLCA and adjacent normal tissues. *P < 0.05, ***P < 0.001. BLCA, bladder cancer; RBscore, ribosome biogenesis related score; IHC, immunohistochemistry.

We assessed expression of RBscore signature genes at the mRNA and protein levels in clinical specimens and cell lines. Consistent with the cohort-based analyses, PIN4, POP4, and PRKDC mRNA levels were higher in T24 cells than in SV-HUC-1 cells and were higher in BLCA tissues than in paired adjacent normal tissues (**Figures 8A, B**). Protein-level differences were also observed in cell-line assays (**Figure 8C**). Given the association of PRKDC with adverse risk in the high-RBscore group, we assessed PRKDC by IHC in paired tumor and adjacent normal tissues from 76 patients who underwent radical cystectomy. PRKDC positivity was observed in 60.5% of tumor samples compared with 14.5% of adjacent normal tissues (*P* < 0.001; **Table 1**), and tumors showed stronger staining and higher IHC scores (*P* < 0.001; **Figures 8D, E**). In clinical analyses, PRKDC positivity was not associated with tumor diameter or lymph node metastasis, whereas expression was higher in stage III than in stage II disease, consistent with an association with progression (*P* = 0.019; **Table 2**).

**Table 1:** PRKDC IHC in MIBC and adjacent normal tissues.

Sample (%)	PRKDC positive	PRKDC negative	*P* value
*MIBC tissues* *(N = 76)*	46 (60.5)	30 (39.5)	
*Adjacent normal tissues (N = 76)*	11 (14.5)	65 (85.5)	<0.001***

***P < 0.001. IHC, immunohistochemistry; MIBC, Muscle-invasive bladder cancer.

**Table 2:** Clinicopathological characteristics of patients stratified by PRKDC IHC status.

Characteristics	*N* (%)		*P* value
	PRKDC positive (*N* = 46)	PRKDC negative (*N* = 30)	
Age			
< *60*	11 (14.5)	12 (15.8)	
> = *60*	35 (46.1)	18 (23.7)	0.201
Gender			
*Female*	17 (25.0)	12 (17.1)	
*Male*	29 (35.6)	18 (22.4)	1.000
Overall Stage			
*Stage II*	16 (21.1)	19 (25.0)	
*Stage III*	30 (39.5)	11 (14.5)	0.019*
T stage			
*T2*	20 (26.3)	20 (26.3)	
*T3*	26 (34.2)	10 (13.2)	0.062
Lymph node metastasis			
*No*	34 (44.7)	26 (34.2)	
*Yes*	12 (15.8)	4 (5.3)	0.253

*P < 0.05. IHC, immunohistochemistry.

## 4 Discussion

BLCA shows marked biological heterogeneity, and routine clinicopathological staging does not fully capture molecular variation relevant to clinical outcome. RiBi is increasingly recognized as a contributor to malignant fitness through its roles in translational capacity, nucleolar stress handling, and adaptation to proliferative and therapeutic pressure, providing a rationale for its investigation in tumors (13, 20). We therefore analyzed RiBi-related transcriptional programs in TCGA-BLCA and validated the prognostic performance of RBscore in the independent GSE13507 cohort. Further analyses related RBscore strata to differences in pathway programs, immune contexture, predicted drug sensitivity, and somatic mutation features. In addition, RBscore signature gene expression was supported in BLCA cell lines and paired clinical tissues, consistent with tumor-associated overexpression.

A prominent feature of our analysis was the recurrent enrichment of RiBi-related terms in BLCA. RiBi programs have been linked to malignant fitness across multiple solid tumors, most notably hepatocellular carcinoma, where increased RiBi has been associated with tumor growth, stress tolerance, and adverse clinical outcome (15). In BLCA, however, evidence has been less cohesive, and RiBi has not routinely been positioned as a unifying biological axis. By intersecting tumor-normal DEGs with a curated RBRG set, we obtained a focused candidate list whose enrichment patterns converged on core steps of ribosome production, including preribosome formation, ribosomal subunit biogenesis, and ribosome assembly. Together, these observations suggest that RiBi-centered programs represent a reproducible transcriptional axis in BLCA and motivate mechanistic studies to define how this axis relates to disease progression.

RBscore links a compact three-gene signature to complementary components of a RiBi-centered tumor program. POP4 is a shared protein subunit of the RNase P and RNase MRP ribonucleoprotein complexes, and the role of RNase MRP in precursor rRNA processing places POP4 within the early RNA maturation steps required for ribosome production (21, 22). PIN4 encodes the metazoan parvulin Par14, which has been identified in preribosomal ribonucleoprotein complexes, co-localizes with the nucleolar protein B23, and delays the processing of precursor rRNAs into mature 18S and 28S species when depleted (23, 24). Together, these observations place POP4 and PIN4 within the RNA-processing arm of RiBi and provide a biological basis for linking RBscore to preribosomal RNA processing. In our data, both genes were upregulated in BLCA cells and tissues but carried favorable coefficients in the prognostic model. This pattern suggests that their contribution may reflect a comparatively ordered RiBi state within tumors rather than greater proliferative drive alone. For PIN4, the inverse association with monocyte infiltration further suggests that this RiBi-linked state may coincide with a less monocyte-enriched microenvironment. By contrast, the absence of a prominent immune association for POP4 suggests a role more closely related to preribosomal RNA processing and function. Overall, these findings suggest that PIN4 and POP4 mark a RiBi program that remains functionally organized rather than one shifted toward a more stress-adaptive or overtly aggressive state.

PRKDC encodes the catalytic subunit of DNA-dependent protein kinase and is best known for its role in nonhomologous end joining. Emerging evidence also suggests that PRKDC may have DNA damage-independent nucleolar functions related to pre-rRNA biogenesis, raising the possibility that genome maintenance and ribosome production are coordinated in specific tumor states (25, 26). In line with this possibility, PRKDC was overexpressed in BLCA cells and tissues, showed tumor-enriched IHC staining, and was associated with adverse outcome, supporting its contribution as the risk-driving component of RBscore. PRKDC expression was positively associated with monocyte infiltration, CD8(+) T-cell infiltration, and CD274 (PD-L1), and negatively associated with the immune-regulatory molecules TNFRSF14 and TNFRSF25 (27, 28). Together, these associations are more consistent with an immune context marked by inflammatory cell presence alongside checkpoint upregulation than with fully effective antitumor immunity. This pattern may reflect a T cell-inflamed but functionally restrained state. A gastric cancer study showing PRKDC-related stabilization of PD-L1 provides a possible mechanistic precedent for this interpretation and further supports a role for PRKDC in promoting T-cell suppression (29). Additionally, the enrichment of TP53 mutations in the high-RBscore group is consistent with the broader aggressive state represented by PRKDC, characterized by concurrent RiBi dysregulation, adaptation to DNA damage, and genomic instability (30–32).

Viewed together, these three genes suggest that RBscore captures more than the summed effects of individual markers. Rather than reflecting RiBi in a purely quantitative sense, the score may index the biological state in which RiBi is operating. At one end, PIN4 and POP4 are more consistent with a relatively ordered RiBi program linked to RNA processing and preribosomal function. At the other, PRKDC points to a more maladaptive state in which RiBi is coupled to DNA-damage tolerance, checkpoint upregulation, and aggressive progression. In this framework, a high RBscore does not simply indicate greater ribosome biogenesis, but a shift from regulated RiBi toward a stress-adaptive and clinically adverse tumor state. This interpretation helps explain the associations of RBscore with survival, immune composition, and broader molecular features. It also provides a biological rationale for the prognostic value of the three-gene model, suggesting that RBscore is not a simple aggregation of unrelated genes, but a compact readout of the balance between RiBi homeostasis and RiBi-linked malignant adaptation in BLCA. This view is further supported by the transcriptomic enrichment of antigen processing and presentation programs in the high-RBscore group, which suggests altered immune contexture as part of this adverse-risk state (33, 34).

More broadly, the present findings support ribosome biogenesis in BLCA as more than a recurrent enrichment signal and instead point to a biologically coherent tumor-state program linked to disease aggressiveness. Prior work in BLCA has more often focused on individual RNA- or RiBi-associated factors, such as NPM1/B23 and PNO1, rather than on RiBi itself as a unifying axis of prognostic heterogeneity (35, 36). Our results begin to narrow this gap. In the current study, RiBi-related genes were reproducibly enriched at the levels of tumor-normal differential expression and functional annotation, and this biology was distilled into a compact three-gene RBscore with external prognostic support.

RBscore captured prognostic heterogeneity beyond any single marker, with clear separation of OS between the high- and low-RBscore groups. Although the AUC was attenuated in the external GSE13507 cohort, this likely reflects the smaller validation sample size and, despite prior standardization, residual cross-platform differences between RNA-seq and microarray data. Even so, the direction of risk stratification and the OS trends remained consistent between the derivation and external validation cohorts, supporting the reproducibility of the main prognostic pattern. The persistence of this separation across major clinicopathological subgroups further suggests that RBscore captures biological variation not fully represented by routine staging alone. RBscore also remained independently associated with OS alongside clinicopathological variables and supported an interpretable nomogram for individualized risk estimation. Inclusion of N stage is biologically coherent, as tumor-draining lymph nodes represent an early interface for dissemination and immune conditioning (37). In this setting, RBscore complements N stage by quantifying tumor-intrinsic programs linked to progression, whereas N stage anchors the model to an established marker of disease spread (38, 39).

From a translational perspective, the practical value of RBscore lies in its compact structure and biological interpretability. Compared with previously reported BLCA expression signatures, including a 7-gene immune-metabolic signature, an 8-gene prognostic signature, and a 12-gene NMIBC progression score, RBscore comprises only three genes in the present analytical framework, which may facilitate implementation on routine platforms such as RT-qPCR or immunohistochemistry (40–42). Its potential value also extends beyond survival stratification. Because RBscore was linked to immune composition, checkpoint-related associations, and TP53 mutation patterns, it may provide a biologically grounded entry point for integrated prognostic assessment and immunobiological interpretation in BLCA.

Additionally, our pharmacogenomic analyses further support the therapeutic relevance of RBscore by linking its signature genes to candidate drug-response signals. Among the nominated compounds, AT-7519 is notable because it is a multi-CDK inhibitor that has entered early-phase clinical testing in advanced cancers, indicating broader oncologic development feasibility (43). In our analysis, however, PRKDC was inversely associated with AT-7519, suggesting that PRKDC may not define a direct target context for this agent and may instead reflect a biological state less compatible with response. By contrast, chelerythrine and SAR-20347 may be of greater interest as exploratory candidates in the present setting, the former supported by broad preclinical anticancer activity and the latter by a mechanistically relevant TYK2/JAK1 inhibitory profile (44, 45). Together, these findings extend RBscore from prognostic stratification toward therapeutic hypothesis generation and provide a rationale for compound prioritization in subsequent BLCA-focused functional studies.

Several limitations merit consideration. First, our analyses relied on public bulk transcriptomic datasets, and prospective validation in larger, clinically annotated cohorts using standardized assays will be required before clinical translation. Second, although integrative bioinformatic analyses prioritized PIN4, POP4, and PRKDC, the functional roles of these RBscore signature genes and their mechanistic links to RiBi and the tumor microenvironment remain to be established. Third, our clinical validation cohort comprised patients with MIBC, which may limit generalizability to NMIBC and earlier-stage disease. Finally, although RBscore retained prognostic value in an external cohort, deployment will likely require prospective calibration and, potentially, integration with additional clinical variables to ensure stable performance across populations and platforms. Together, these considerations motivate further validation and mechanistic work to refine RBscore and define its utility in clinical risk assessment.

## 5 Conclusion

In summary, this work delineates RiBi-linked transcriptional programs in BLCA and defines a three-gene RBscore that stratifies OS across cohorts and is associated with pathway enrichment, immune contexture, and complementary drug-response and genomic signals. These results provide a biologically grounded framework for risk stratification and generate testable hypotheses for biomarker development and therapeutic exploration.

## Data Availability

The datasets presented in this study can be found in online repositories. The names of the repository/repositories and accession number(s) can be found in the article/**Supplementary Material**.
