# Supplementary material for: Ribosome biogenesis programs define a three-gene RBscore with prognostic relevance in bladder cancer
Source: Front Immunol. 2026 Apr 16;17:1810132. doi: 10.3389/fimmu.2026.1810132 (PMC13128572; doi:10.3389/fimmu.2026.1810132)
Supplement: Supplementary file 1 [file DataSheet1.docx]

**Ribosome biogenesis programs define a three-gene RBscore with prognostic relevance in bladder cancer**


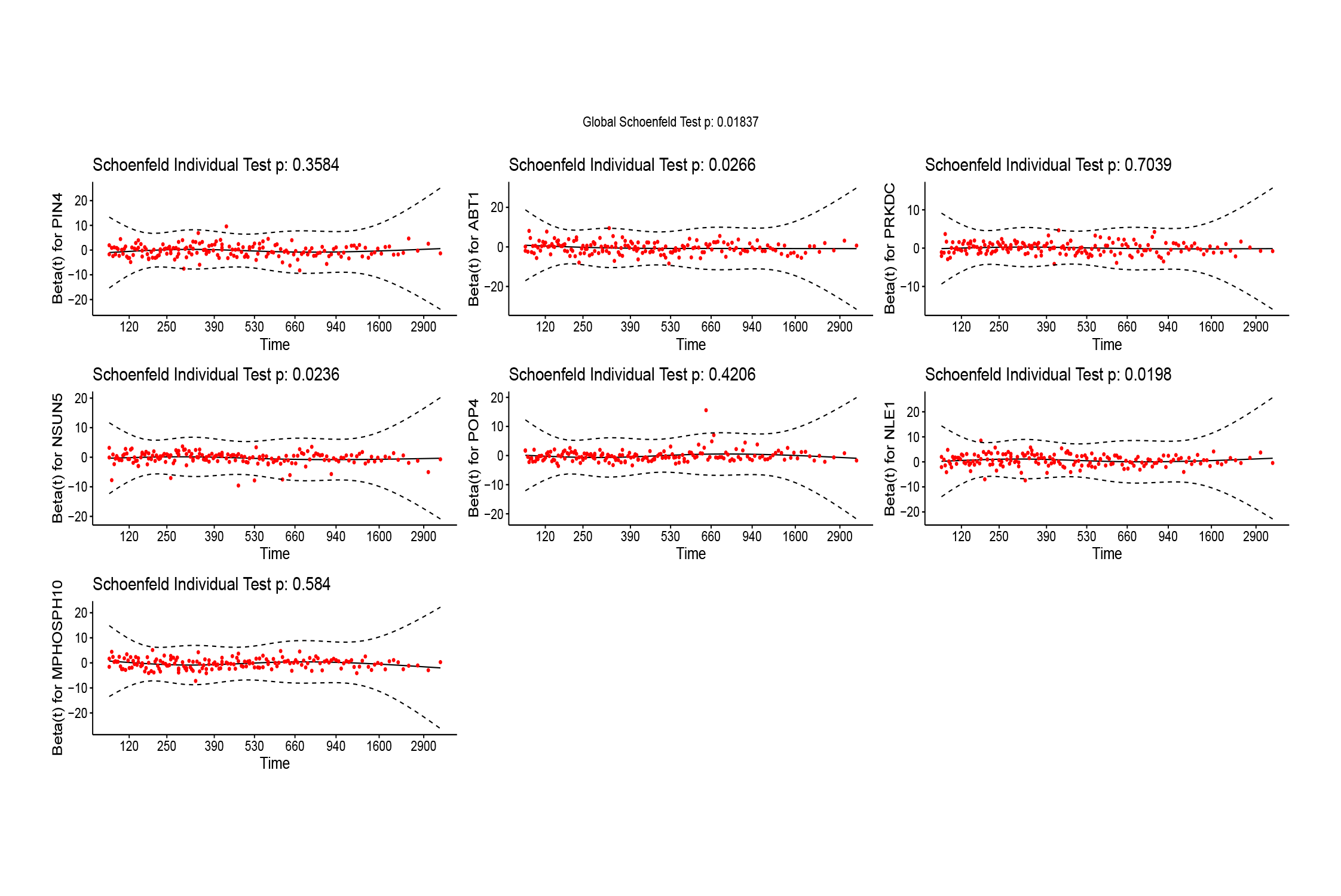


**Supplementary figure 1. PH assessment of univariate Cox-significant genes.** Schoenfeld residual plots are shown for genes identified by univariate Cox regression. PH, Proportional hazards.


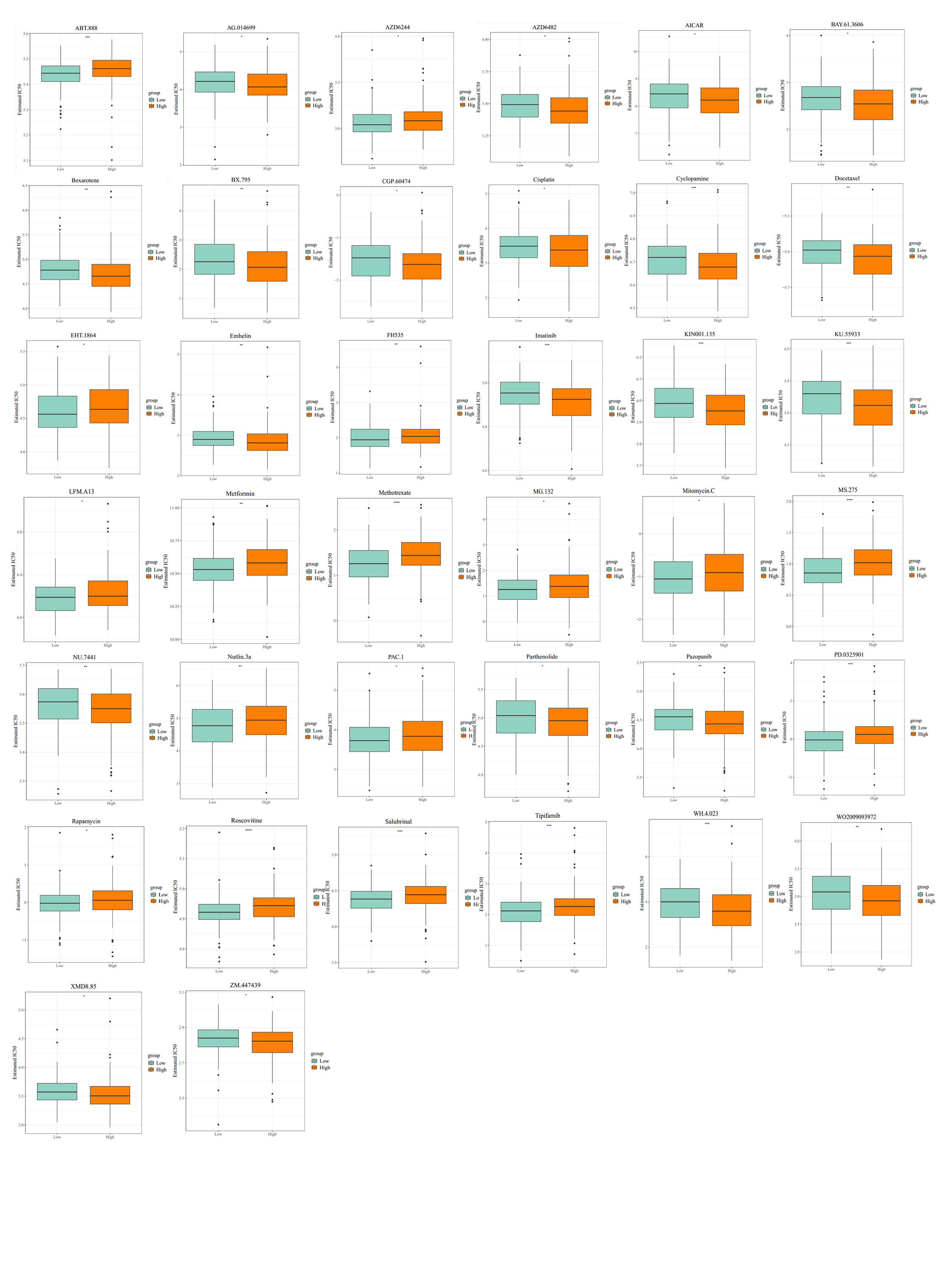


**Supplementary figure 2. Predicted drug sensitivity differences across RBscore groups.** Box plots show inferred IC50 values for 38 compounds in the training cohort, comparing the high and low RBscore groups. RBscore, Ribosome biogenesis–related score.


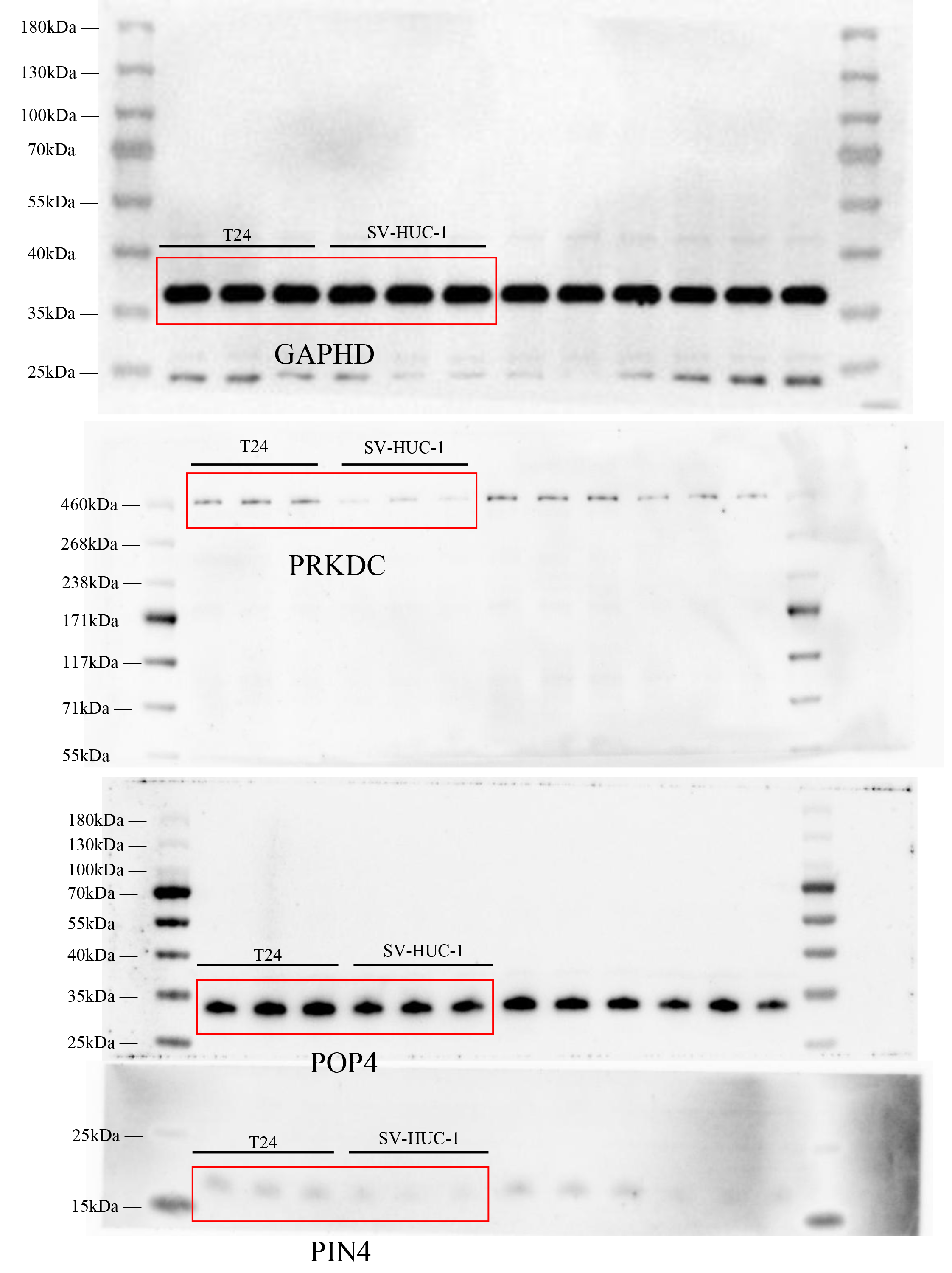


**Supplementary Figure 3. Full-length, uncropped western blot scans corresponding to Figure 8C.** Full-length, uncropped scans are provided for GAPDH, PRKDC, POP4, and PIN4 in SV-HUC-1 and T24 cells (molecular weight markers in kDa are shown; lane order matches Figure 8C). These proteins were detected on separate membranes prepared from parallel gels run in the same experiment using the same lysate set and identical lane order, with each membrane probed for a single target. Red boxes indicate the regions cropped for presentation in Figure 8C. As in Figure 8C, the GAPDH blot is reproduced in the PRKDC/POP4/PIN4 panels for illustrative purposes and this reuse is explicitly disclosed here.

| **No.** | **Primer Name** | **Sequence（5' to 3'）** |
| --- | --- | --- |
| 1 | PIN4-F | GGCTCAAGGTCCCAAAGGTG |
|  | PIN4-R | ACCCTCTGGTCATCCAACCC |
| 2 | POP4-F | CACGCAGCCACAGATGATTC |
|  | POP4-R | TGGGGATAACTTTCAGGCGG |
| 3 | PRKDC-F | ATGTCCCAAGAGGAGAAGGC |
|  | PRKDC-R | TCAGTACGATTAGCGCCCTT |
| 4 | ACTB_F | CTGCCCCAGAATGGTTTTTA |
|  | ACTB_R | TGAGGGACTTCCTGTAACAATGCA |

**Supplementary table 1. Primer and siRNA sequences used in this study.**

| **Variable** | **Coef** | **HR** | **HR.95L** | **HR.95H** | **P value** | **PH** |
| --- | --- | --- | --- | --- | --- | --- |
| ***Age*** | 0.0237 | 1.0240 | 0.9984 | 1.0502 | 0.0662 | 0.7328 |
| ***Sex*** | -0.4601 | 0.6312 | 0.3765 | 1.0585 | 0.0811 | 0.4785 |
| ***Stage*** | 0.6038 | 1.8290 | 1.3056 | 2.5623 | 0.0004 | 0.2773 |
| ***T*** | 0.5280 | 1.6956 | 1.1785 | 2.4396 | 0.0044 | 0.5187 |
| ***M*** | 0.9380 | 2.5549 | 1.0192 | 6.4045 | 0.0454 | 0.6466 |
| ***N*** | 0.4548 | 1.5758 | 1.2228 | 2.0306 | 0.0004 | 0.2883 |
| ***RBscore*** | 0.0504 | 1.0517 | 1.0417 | 1.0619 | 0.0000 | 0.3709 |

**Supplementary table 2. Univariate Cox analysis of OS for clinicopathological features and RBscore.** Univariate Cox proportional hazards regression was used to assess associations of age, sex, overall stage, T stage, N stage, M stage, and RBscore with OS in the training cohort. P values and PH test P values are shown. OS, overall survival; Coef, coefficient; HR, hazard ratio; PH, proportional hazards.

| **Drug** | **Gene** | **PDBID** | **Affinity(kcal/mol)** |
| --- | --- | --- | --- |
| ***SAR-20347*** | PIN4 | 3UI4 | -9.3 |
| ***Chelerythrine*** | POP4 | 6AHU | -7.5 |
| ***AT-7519*** | PRKDC | 5LUQ | -8.4 |

**Supplementary table 3. Binding energies of active compounds corresponding to RBscore signature genes.**
